# Address of the President on the Philosophy of Homœopathy

**Published:** 1881-07

**Authors:** P. P. Wells

**Affiliations:** President; Brooklyn, N. Y.


### THE PHILOSOPHY OF HOMŒOPATHY.

ADDRESS BEFORE THE INTERNATIONAL HAHNEMANNIAN ASSOCIATION, BY P. P. WELLS, M. D., PRESIDENT.

GENTLEMEN: At the meeting of the American Institute of Homœopathy, at Milwaukee, in June, 1880, this association was organized, as was said, for the accomplishment of a definite purpose, viz.: the advocacy and illustration of *pure* homœopathy. In the use of this word *pure*, it was understood to antagonize that which is partial by that which is complete, and that which is mixed by that which stands in its grand simplicity in the "*Organon of Homœopathic Medicine*," as given to us by the author of "The Science of Therapeutics." It was understood that those who then combined for this purpose held the science, as there contained, as embodying a law of therapeutics complete in its application to the needs of all healing, and universal in its adaptability to the relief of all pains and sicknesses within the pale of salvability. They declared this law to be, as there taught, the law of similars, and combined with this the declaration, that certain corollaries were a necessary part of its practical application in all clinical duties. These were "*the totality of the symptoms, the single remedy, the minimum dose of the dynamized drug.*" These associates declared that this law, with these corollaries, together constitute the *pure* homœopathy they proposed to advocate and illustrate. That these corollaries are a necessary integral part of the true and best practical administration of the law of similars. By this it is supposed they intended, and we believe rightfully, to antagonize that partial homœopathy which accepts the law of similars, or professes to, and then founds its therapeutics on generalizations, which it calls "*pathological conditions*," and for these, often only hypothetical, gives crude drugs, and perhaps in massive doses, claiming, while so doing, to be obeying the law of therapeutics which the "Organon" proclaims, which at the same time is admitted to be nature's law of healing. This partial homœopathy stops at the "*similar remedy.*" It will go no farther. It might almost as well not have gone at all. It admits, in order to a cure under the law, the necessity of the similar remedy, which is well as far as it goes. But if, when asked what is your remedy to be similar to, in order to a compliance with the demands of our law of therapeutics, it be answered, to the "pathological conditions," we reply: How are you to gain a knowledge of these? If there be any other medium through which a knowledge of these can be obtained than that of the perceptible symptoms of the case, it must be the imagination of the prescriber, and this it may as well be understood, homœopathy will not accept as a sufficient guide. Hence, even the partial homœopathist, if logical, is compelled to accept the first corollary of the law—"*the totality of the symptoms.*" And when compelled to fall back on these, it is not perfectly clear how the "pathological condition" idea adds to the efficiency of their guidance to the right selection of the similar remedy. For it should be borne in mind that the similarity to be sought for and required is in the likeness of the effects of some drug to this totality, and not at all to any supposed condition, which, from the very nature of things, must be in a great degree a matter of pure conjecture. That homœopathy has to do wholly with ascertained fact, and not in any wise with any man's conjecture, however ingenious. The acceptance, then, of the first corollary, would seem to be a necessity, even to the partial homœopathist, if he loyally and logically accepts the therapeutic law as authoritative. By the same force he will be compelled to accept the second. The law requires at his hand, for the cure of the sick, the remedy which in its effects is *most like* the phenomena of the case to be treated. Now as no two drugs are identical in their action on the living organism, it follows that of any two drugs having developed similar symptoms to those of the case to be treated, one of them must be more like to the symptoms of the case to be cured than the other, and therefore it is the required remedy for the case; and so it becomes self evident, that the other is not, and for the reason that it is not most like. If one be most like, the other cannot be, for no two are identical. Logic will compel the partialist to the acceptance of this second corollary also. The third corollary, "*the minimum dose of the dynamized remedy,*" though not made a necessity by the logical requirements of the law, is fully sustained by reason and the records of long practical experience. By "the minimum dose," we understand, the least quantity of the drug the cure requires. As the object for which it is given is the cure, all that is given more than this requires is always superfluous, to say the least, and may be, and often is, a source of mischief—therefore reason sanctions and requires this minimum. That the dose be not only the least that will effect the cure, but that it be also a "potentized" dose, it is believed is required in order to the realization of the best practical results from the administration of our therapeutic law. This is accepted on the testimony of those who have tried it extensively, and compared the results of the use of potentized and unpotentized drugs. They say their experience fully sustains the greater curative power of the former. It is self-evident the value of this corollary must be decided by this practical tribunal, *i. e.*, by the experience of those who have practically tried both the dynamized and crude drug, and compared the results of the use of each. It is just here that *a priori* reasoning of those who have made no such trial has no place, and cannot, with reasonable men, have the least weight as against the testimony of those who have. Now it is homœopathy as here expressed by the law and these corollaries, that this association proposes to advocate and illustrate; and this in order to secure better practical results from the administration of the law for the cure of the sick. It is this for which we are chiefly concerned, and for which, as an association, we exist. Then it should be our first object to comprehend clearly what a right administration of this law requires of the prescriber. In order to this, let us see what is contained in the expression, "*totality of symptoms.*" As prescribers, it is with these we have our starting point. Until we have these in our possession, we have no concern with the other factors of the problem we are about to try to solve. A right understanding of this fundamental expression is necessary before we can take the first step in a true homœopathic prescription. "*Totality of symptoms*"—what does it mean here? All the symptoms of the case, is it answered? It means this and more. The "totality" here means not only the sum of the aggregate of the symptoms, but also this other and most important fact of all, in true homœopathic prescribing: the totality of each individual symptom of the aggregated group. A single symptom is more than a simple fact; it is a compound, made up of a fact, with its history, its origin, progress and conditions attached. If it be a cause of suffering to the patient, then in it are included all the circumstances of its aggravation or melioration; as to time of its greatest intensity, position, motion, rest; how affected by eating, drinking, or the performance of any bodily function; how affected, if at all, by different mental emotions; or by any other cause of increase or relief of suffering. All this is included in the "*totality*" of each single symptom, and without all this the prescriber is ignorant of the intimate nature of the symptom for which he is to find a simillimum. He is to know all this of each and every symptom of his case before he is prepared to take the first step in his search for the like which cures; and this for the reason that it is likeness to these secondary elements of the symptoms, which only are declarative of their intimate nature, to which he is to find likeness in the recorded action of the drug he is to select. Having a clear knowledge of all the symptoms of his case, as here set forth, the prescriber is prepared to use a similar knowledge of the agents he is to employ for his cure.

By a similar knowledge, we mean a knowledge of the totality of the symptoms which the drugs he employs have been ascertained to have produced in the healthy, living organism. By "totality" here, we mean not only all the disturbances of function and sensation these have produced, but as in the case of the results of the impression of the morbific cause (symptoms of disease), we are here to take into account all the modifying circumstances and conditions which produced, accompanied, aggravated, or relieved these drug sufferings in the prover. These will be found duly set forth in every proving of a drug which is worthy of respect, or of being received as a proving. These are the facts which give to a proving almost its entire value; and it is the absence of these in many of the modern, so-called, provings which stamps upon them their character of utter worthlessness for the purposes of the true homœopathic prescriber. It is chiefly in these elements of morbific and drug action that the likeness is to be sought and found which cures. The relationship which constitutes the drug a curative of the disease, exists in the similar nature of these concomitants of the morbid and drug action. Hence the absolute necessity of a thorough understanding of these concomitants of the two factors of the problem the prescriber is to solve, *i. e.*, to the finding the simillimum which cures. It is found, and only found, in the likeness of these concomitants the one to the other.

But the partialist says, what is the use of all this trouble? I accept the law of similars and that is enough for me. I am quite satisfied with the results of the practice I have founded on the general acceptance I have given to this law. He believes the law of the similars is nature's law of cure, and he does not feel called upon to go farther and seek a more definite faith. If, when he says, I believe in the law of similars, he be asked, what are the similars which constitute the basis of your law, and where are they to be found? the answer, if he stops at the general acceptance of the law, is not very obvious. If left there, his position is not very different from that in the old-school fog, from which an intelligent reception of the law and its necessary corollaries, with a clear knowledge of the elements gained by a proper study of the disease and its curative as we have endeavored to set them forth, can alone give him complete emancipation.

If without these he ever effects a true homœopathic cure, it would seem that it must have resulted from pure accident. But in your declaration of principles, which you affirm are taught in the "Organon," you go still further than this, and say that the drug to be given for a cure must, when found, not only be given in a minimum dose, but in dynamized form. The first part of this has already been considered, and we have seen the self-evident wisdom of it; because the only object of giving the drug is to effect a cure; to give more than is needful for this purpose is always useless, and may be, and often is, greatly mischievous. But that the dose be dynamized is not a logical necessity of an acceptance of the law of similars, as are the totality of the symptoms and the single remedy; neither is its necessity self-evident, as is that of the single remedy and the minimum dose. This part of the declaration as to the dose is not the offspring of logic or induction at all; it has never been inferred from any other fact or facts; it has been of a more sure parentage—of experience and observation, and of these alone. It is not too much to say that the observations of an enlightened experience fully sustains the existence and value of this, her interesting child. When we say *enlightened experience*, we mean experience enlightened by a clear perception of homœopathic philosophy, and guided in all its practical endeavors by this—it may be added, that all the utterances of all those who have had no such experiences, in antagonism to practice with dynamized remedies, are, before the testimony of those who have had, in its favor, worth no more than the chaff which the wind drives away. The claim to our acceptance of the dynamized dose in our practical duties, rests then wholly on the testimony of those who have tried it, and who know the truth of that which they affirm. The history of the dynamized dose, as contained in the testimony of these witnesses, declares its superior curative power, as compared with doses of crude drugs. It should be borne in mind when considering the statements of these witnesses, that most of them are the more competent to give us testimony worthy of our regard, for the reason that they have practically tried both kinds, and have found greater curing power in that which is dynamized.

In addition to this, we believe it may be safely affirmed that the great majority of the most remarkable cures effected by the homœopathic administration of drugs, have resulted from the dynamized dose. Even Wurmb, prejudiced as he confessed he was against the claim of a superior curing power in the dynamized dose, was constrained to say, when he saw the results of his ten years' experiments declaring the superior curing power in the higher dynamizations, that he could see no good reason for doubting, in the light of these results, that his practical success would have been greater if he had used higher dynamizations. How, indeed, could it have been otherwise with an honest man when these showed him that in proportion as he gave higher dynamizations, the proportion of those cured to those treated was greater—that the cures were effected in a shorter time, and that the average duration of the period of convalescence was abridged in a notable degree; thus showing, not only more patients were cured by the higher dose, but that the cures were more perfect! Who, after this showing, if he be honest, can doubt, more than could Wurmb, that he could have cured more, and still more perfectly, if he had employed still higher numbers in his experiments! Wurmb was an honest man, and did not fear to testify against his prejudices when truth required this at his hands.

The above, then, is what we understand by a complete homœopathy as opposed to that which, though so-called, is only partial, because while it professes acceptance of its law of similars, it rejects its logical and necessary corollaries, which are virtually and practically a part of the law itself, and are indispensable to its intelligent and best practical administration. It is also given as a whole which needs no aids from without the law, in order to the attainment of the greatest practical successes in treating the sick. It knows no needs of these, when it is administered as above set forth, and whenever and wherever these are interjected into the homœopathic treatment of the sick, they constitute the mixture which is so great a blemish, and often so great an injury, and even so great a danger to the health and life of the patient, and which the declaration of principles given by this association at the time of its organization was intended to antagonize. It was intended then to say, and we here repeat, that the simillimum in our case needs no aids, and tolerates no interference with its specific action. To attempt this by any means which change the true homœopathic treatment of a case, to a treatment which is mixed, we have no hesitation in declaring a senseless movement, and wholly without excuse.

It has been objected to the above view of homœopathy and its practice, that the one is obscure because it is so largely concerned with subjective phenomena, and these are exceedingly liable to mislead the patient and deceive the prescriber, because they are wholly beyond the reach of his senses; and that the other is too difficult for every prescriber in his every-day routine of duty. The reply to both these objections is, that the prescriber has to do with *all* the facts in his case, and that to neglect to gain a knowledge of any one or more of them because they cannot be brought to the cognition of his own senses, or because the mastery of it is difficult, is to play the part of a poltroon, and not at all that of a true homœopathic prescriber. Subjective phenomena are facts, and not only this, they are *the* facts with which the true prescriber has most largely to do. It is his business to see to it, that if these are liable at times to mislead the patient, they are not to be permitted to deceive him, and till he is equal to protecting both his patient and himself from deception from these, to his patient's injury, he is not a master of the business he has undertaken. The difficulty of gaining a possession of the totality of the symptoms, as above represented, *i. e.*, the totality of each individual symptom, as well as of the aggregate of the group, is freely admitted—if it be not the most difficult of human duties, it is by far the most difficult part of practical prescribing. Finding the most similar medicine after the symptoms in their totality are known, is comparatively easy. Said a great master in our school to me: "When the symptoms of a case are fairly and clearly drawn out, the case is more than half cured." This is true; hence the importance of these being rightfully known in their totality; without this, there is no such thing as homœopathic prescribing. Then the difficulty of mastering a knowledge of the totality of the symptoms of the drug agencies we are to employ, in the same manner and kind as we have found necessary in the case of those of the disease, adds greatly to the labors of the prescriber. Said one to me, who was not a master in our school, though he professed to belong to it, when the necessary process of arriving at a specific prescription was explained to him: "I do not know enough to make a prescription like that." This was no doubt true. But what then? Should a man continue before the community, professing to do for it, in a matter so important as curing its sicknesses by specific prescribing, that which, privately and truly, he confesses he does not know enough to do? Such a one should remember no one ever knew enough for this till he had learned it, and that no one ever learned it by other means than hard work—long continued and incessant. This is the only cure for that most troublesome of all diseases to the prescriber—don't know enough. By proper diligence, he may come to know more—even enough to enable him to make a true specific prescription.

Then as to the use of the potentized remedy. This has been objected to as a something so very shadowy and evanescent as to be beyond apprehension or control, by whatever means employed, in the prosecution of a knowledge of other sciences than that of scientific prescribing. They are not amenable to the senses, these dynamizations, nor to the perception of chemical tests, however delicate and sensitive these may be. The objection would be a valid one if the object of the prescriber were to make a definite impression on the senses of the patient or his friends, or to demonstrate the truth of any question in chemistry or physics. As it is neither of these with which the prescriber has to do, the objection has no standing in the case. But if there be any one, who from whatever of prejudice or ignorance is still disposed to urge this objection, it is a sufficient answer to say, that however the senses or scientific tests may fail to detect the presence of the potentized similar remedy, because they have no relationship to it, there is another and more subtle agent, which does not fail to recognize and respond to its presence whenever the two are brought into a certain relationship to each other. The sensibility of the sick organism never fails to respond to the presence of the similar specific curative, even though this be very highly potentized; indeed, the best experience and observation have abundantly testified that the curative response of the sick powers is all the more complete because of this potentization. The difference of result between an appeal to this test and that to the senses, or to the tests of physical science, is just because in the one case the God of nature established a relationship to endure as long as pains and sickness afflict our race; and in the other, there is no relationship whatever.

And then if the objection be still to the potentized similar remedy because of its shadowy and evasive form, it is a sufficient reply, that this is no more true of the potentized curative than, in a multitude of cases, it is of the morbific cause which has produced the sickness to be cured. The great bulk of the talk of germs, or living organisms, as such causes, now so fashionable, may very safely be dismissed to the limbo which has swallowed so great a multitude of worthless hypotheses of the past. This is the more a pity because, if true, the problem would only be to find the agent which would destroy the germs, and then we should have the demonstration of the truth of the proverb which told us long ago that prevention is better than cure—this would be even better than pure homœopathy. The germs of yellow fever—who has not heard of them? Were they not living organisms? And who has not been told of carbolic acid as the germicide? In other words, the great disinfectant? And who does not remember the floods of this acid used in the late great epidemic of that fever in the Mississippi Valley, before the wise doctors found out that, though it might be death to germs, it did not disinfect.

One word more as to this matter of dynamization. You, gentlemen, who have given his name to your association as that by which you will be known, will remember that this fact, the discovery of which belongs exclusively to Hahnemann, is that which constitutes the crowning glory of his life and labors. He is often spoken of as the discoverer of the law of similars, which he was not. This had been recognized by the most observing minds of antiquity centuries before Hahnemann's birth. He only gave to this previously and partially received philosophy a more general recognition and acceptance. In that he took his first hint of the law from the disclosures of his own experiments, he may be said to be, in a certain sense, its discoverer. But his chiefest glory was the discovery of the fact of dynamization, which has given to us knowledge of new power in drugs, and the possession of many of our most important healing agents. But for this discovery, we should never have had in the list of those of greatest value, Sepia, Silicia, Calcarea, Carbo. veg. Graphites, Nat. mur., and many others, to be deprived of which now, would be to rob us largely of our most valuable resources. This discovery and his philosophy of chronic diseases, now too much neglected, are the two great facts which characterize the life of the master as a discoverer in the field of practical medicine. There may have been a claim to divide the honor of this last; but as to the first, it was Hahnemann's alone, and its value and importance are enough to crown the memory of any man's life with immortality.

Gentlemen of the International Hahnemannian Association: In what I have said, I have endeavored to give the constitution of the homœopathy of Hahnemann as he has given it to us in his "Organon of Homœopathic Medicine." Do you accept this statement as a representation of that for the advancement of which you have joined in this association? Are the principles we have now discussed those which in your associated capacity you propose to advocate, and in your practice to illustrate? If the affirmative of this question be true, then another is ready to follow. By what means do you propose practically to prosecute this advocacy? and what are the grounds on which you can base a reasonable expectation that your endeavors will result in bringing others to the acceptance of these principles which to you are so precious? Do you say, we rely on the power of truth to make its way to the conscience and acceptance of reasonable men, when brought to their notice, simply because it is truth? This is not enough, as the world, even the reasonable men in the world, is now made up. It implies life of conscience, absence of prejudice, and an intelligence equal to the appreciation of these principles in all their purity and extent. It has not been because these principles were not true, that now they have not universal acceptance with men—even with men who mean to be reasonable. The difficulty has not been lack of truth; it has not been the fault of the principles, but of the men, who, whatever excellencies they may possess in all things where these principles are not concerned, are now the living representatives of those of old, of whom it was said: "Ye will not come to the light." The difficulty, then, is not in a want of proof of the truth of these principles, but in the will of the objector or sceptic. And when the question is of means by which to prosecute a successful advocacy of these principles, with a view to their extended acceptance, it must be understood to refer to means capable of overcoming both prejudice and will, and we confess that to our minds the answer to the question is difficult. It cannot be found in violence of attack—that never convinced a man against his will yet. It is not argument that these men need; if it were, we would give it; but they have had argument already, *ad plenam*, till now there is little left of this which is new for them. And then argument never yet changed a perverted will. It is not a want of knowledge that is to be supplied by any teachings which this association is capable of supplying that constitutes the difficulty to be overcome—the great difficulty is, men will not know. They will not listen to the instruction you may be willing to give. Neither is it to be removed by controversy—this only ends in confirming each side in his own opinion. It never yet changed a perverted human will, nor brought down false pride to the level on which the voice of wisdom can be heard. And before a partialist or a mixed prescriber can be brought to the acceptance of the truth in its simplicity, both these mighty obstacles are to be overcome. In this respect, in their relation to complete homœopathy, they differ little, if at all, from the one of the old-school who has only will, prejudice and pride to sustain him in his rejection of the whole matter. It may be said of the three classes alike what Hudibras said of an individual:

"A man convinced against his will,
Is of the same opinion still."

What then are the members of this association to do, the results of which will justify their existence as an associated body? We know of but one thing, and that is, *work*—earnest, honest, incessant work. Not work upon partialists, mixed, or old-school men, but on the elements of sickness, that a knowledge of them in their totality, as we have shown to be necessary, in order that an intelligent treatment of them practically may the more readily be obtained when needed; and upon the materia medica, that its elements may be mastered in the same detailed totality, in order that when the simillimum for a cure is needed it may be more readily found, and applied with that certainty of assurance of which guessing makes no part. Work of this sort, persisted in, will by and by mature a power greater than any argument, however masterly, or than any controversy, no matter with what earnestness it may be waged. Work of this sort will in time, by its results, so demonstrate to the public mind the superiority of the pure practice of the homœopathy we advocate, over that which is partial or mixed, as well as over that of the old-school, that these gentlemen, recognizing the education the public has thus received, and at the same time the confidence of the public they themselves have lost, are not in the least danger of neglecting to make haste to claim their share of the honor a numbering with those thus diligent is sure to confer. Thus, and thus only, can the interests of true homœopathy be advanced, and the objects for which this association was organized be secured. And in the results of such work only will true homœopathy find its just illustration to which you, gentlemen, by your association, stand pledged to give to the world.

